# Pan‐NLRome analysis uncovers genetic diversity and evolutionary dynamics among rice, maize and sorghum

**DOI:** 10.1002/tpg2.70257

**Published:** 2026-05-23

**Authors:** Yanbo Wang, Tashi Dorjee, Yinzi Wang, Di Peng, Lang Chen, Chuanzheng Wei, Shichao Sun, Mingyu Duan, Hui Li, Adrian Hathorn, Emma Mace, David Jordan, Xun Wu, Xiaocui Chen, Yongfu Tao

**Affiliations:** ^1^ State Key Laboratory of Tropical Crop Breeding, Shenzhen Branch, Guangdong Laboratory of Lingnan Modern Agriculture, Key Laboratory of Synthetic Biology, Ministry of Agriculture and Rural Affairs, Agricultural Genomics Institute at Shenzhen Chinese Academy of Agricultural Sciences Shenzhen China; ^2^ Zunyi Academy of Agricultural Sciences Zunyi China; ^3^ Queensland Alliance for Agriculture and Food Innovation (QAAFI) The University of Queensland, Hermitage Research Facility Warwick Queensland Australia; ^4^ Queensland Department of Primary Industries (DPI) Hermitage Research Facility Warwick Queensland Australia; ^5^ Institute of Upland Food Crops Guizhou Academy of Agricultural Sciences Guiyang China; ^6^ Ministry of Agriculture and Rural Affairs Key Laboratory of Crop Genetic Resources and Germplasm Innovation in Karst Region Guiyang China

## Abstract

Nucleotide‐binding leucine‐rich repeat (NLR) genes constitute one of the largest families of plant immune receptors and are central to crop disease resistance. Rice (*Oryza sativa* L.), maize (*Zea mays* L.), and sorghum (*Sorghum bicolor* (L.) Moench) are major crops underpinning global food security, and enhancing their immunity is critical for yield stability. Here, we conducted a comprehensive analysis of NLR repertoires across 75 genomes from these three grass crops, identifying 24,944 genes that exhibit extensive intra‐ and interspecific variation. The crops share broadly comparable domain architectures of NLR genes, despite their differences in gene number and genomic clustering pattern. We detected 68 integrated domains, revealing high diversity and pronounced lineage specificity. The pan‐NLR profiles of the three crops were generally similar, with strong lineage‐restricted expansion of NLR gene families. Cross‐species comparisons revealed distinct proportions of common gene and lineage‐specific genes between the crops, suggesting divergent evolutionary trajectories. Widespread presence/absence variation of NLR genes was identified within each crop, with an enrichment in clustered loci. NLRs, especially clustered ones, were significantly enriched in disease‐resistance quantitative trait locus (QTL) hotspots, supporting their critical role in crop disease resistance. Notably, conserved *Rp1‐D* clusters co‐localized with resistance QTLs in the three crops, underscoring functional conservation and the translational potential of cross‐species NLR studies. Collectively, these findings provide new insights into the genetic diversity and evolutionary dynamics of NLRs in three major grass crops and provide a valuable genomic resource for enhancing crop disease resistance.

## Plain Language Summary

Plant diseases reduce crop yields, so improving natural disease resistance is important for stable food production. Rice, maize, and sorghum rely on immune receptor genes that help plants recognize and fight pathogens. By comparing these genes across 75 genomes, we found nearly 25,000 immune receptor genes and saw that their numbers and locations vary widely within and between the three crops. Many of these immune genes were not shared by all varieties: some lines carried them, while others did not. When these genes appeared, they were often packed together in small stretches of DNA, and these stretches repeatedly matched regions that plant geneticists have already tied to disease resistance. We also found several gene clusters that are shared across rice, maize, and sorghum in those resistance regions, pointing to promising targets that breeders can prioritize and potentially translate from one crop to another.

**Abbreviations**
CN: coiled‐coil nucleotide‐binding
CNL: coiled‐coil nucleotide‐binding leucine‐rich repeat
CNLX: coiled‐coil nucleotide‐binding leucine‐rich repeat with integrated domain
CNX: coiled‐coil nucleotide‐binding with integrated domain
GWAS: genome‐wide association study
ID: integrated domain
LTR: long terminal repeat
NB‐ARC: nucleotide‐binding adaptor shared by APAF‐1, certain R genes, and CED‐4
NLR: nucleotide‐binding leucine‐rich repeat
NLX: nucleotide‐binding leucine‐rich repeat with integrated domain
NX: nucleotide‐binding with integrated domain
OG: orthogroup
PAV: presence–absence variation
QTL: quantitative trait locus
QTN: quantitative trait nucleotide
RNL: RPW8‐type nucleotide‐binding leucine‐rich repeat
RVT: reverse transcriptase
SNP: single‐nucleotide polymorphism
TE: transposable element
TIR: Toll/interleukin‐1 receptor
TNL: TIR‐type nucleotide‐binding leucine‐rich repeat

## 1 INTRODUCTION

Plant diseases and pests contribute significantly to global crop yield reductions, estimated at 20%–40%, posing a substantial threat to food security (FAO, 2021). Resistance (R) genes are essential for mitigating yield losses, as they mediate immune responses that enable plants to combat pathogens and pests. Among the various classes of R genes, the nucleotide‐binding leucine‐rich repeat (NLR) gene family is the most extensive and predominant across many plant species. NLRs are characterized by conserved domain structures that facilitate pathogen recognition and signal transduction. Investigating the genetic diversity and evolutionary dynamics of NLR genes offers valuable insights that could improve their integration into breeding programs, ultimately contributing to the development of resistant crop varieties and sustainable agricultural production.

NLR genes typically contain three canonical domains: the NB‐ARC (nucleotide‐binding adaptor shared by APAF‐1, certain R genes, and CED‐4) domain, which mediates signaling; a C‐terminal leucine‐rich repeat domain, involved in pathogen recognition; and an N‐terminal signaling module, which executes effector‐triggered immunity (Jones et al., 2016). However, functional NLRs can also be formed with a subset of these domains (Maekawa et al., 2011). Based on their N‐terminal motifs, plant NLRs are classified into three major subclasses: TIR‐type NLRs (TNLs), which contain a Toll/interleukin‐1 receptor domain; CC‐type NLRs (CNLs), which contain a coiled‐coil domain; and CCR‐type NLRs (RNLs [RPW8‐type nucleotide‐binding leucine‐rich repeat]), which feature an RPW8 (Resistance to Powdery Mildew 8)‐like coiled‐coil domain. RNLs primarily function as helper NLRs, collaborating with TNL sensors to enhance immune responses (Castel et al., 2019; Jubic et al., 2019; Lüdke et al., 2022). The distribution of these NLR subclasses varies across species. Comparative genomic analyses have revealed the coordinated loss of TNL and RNL subclasses in monocots (E. L. Baggs et al., 2020; Guo et al., 2025; Lapin et al., 2019).

In addition to canonical domains, integrated domains (IDs) represent noncanonical modules through which NLRs expand their recognition capacity and acquire novel functions (Cesari et al., 2013). These domains are thought to originate from host proteins targeted by pathogen effectors and act as baits or mimics to broaden pathogen recognition (E. Baggs et al., 2017). The repeated emergence of NLR‐IDs across multiple plant lineages suggests that domain integration is a recurrent strategy during long‐term host‐pathogen coevolution (Liu et al., 2021). IDs not only highlight the structural plasticity of NLRs but also provide a valuable framework for understanding their functional diversification, lineage‐specific adaptation, and the evolutionary dynamics of the pan‐NLRome (Prigozhin et al., 2025; Wang et al., 2025).

The genetic diversity and evolutionary dynamics of NLR genes have been extensively studied due to their pivotal role in plant–pathogen interactions (Jin et al., 2024; Tirnaz et al., 2020). The conserved domain structure of NLR genes has enabled the cloning and mapping of resistance gene analogs, leading to the identification of candidate loci for disease resistance in major crops (Bayer et al., 2019; Dolatabadian et al., 2020; Mago et al., 1999; Seah et al., 1998; Xiao et al., 2007). The advent of reference genomes has facilitated the genome‐wide characterization of NLR genes across plant species (Lan et al., 2025; E. Mace et al., 2014; Martin et al., 2023; Meyers et al., 2003; Wei et al., 2024). NLR genes are frequently organized in clusters, a genomic arrangement that contributes to copy‐number variation (Alamery et al., 2017; Ding et al., 2020; Gan et al., 2025). Intraspecific analyses have revealed substantial diversity in NLR genes, highlighting their critical role in crop domestication and adaptation (E. S. Mace et al., 2013; Yang et al., 2008). Cross‐species comparisons have demonstrated that NLR repertoires can vary by an order of magnitude among different plant lineages (E. Baggs et al., 2017; Kan et al., 2024). The incorporation of IDs represents a key evolutionary strategy for NLR genes to interact with pathogen effectors (Chia et al., 2024). Recent characterization of the *Arabidopsis thaliana* (L.) Heynh pan‐NLRome, using targeted enrichment sequencing of 46 representative accessions, revealed significant variation in NLR domain architectures across individuals (Van De Weyer et al., 2019). Furthermore, the development of pan‐genome resources for crop species has enabled the comprehensive examination of the Pan‐NLRome in a range of species, including maize (*Zea mays* L.), *Brassica rapa* L., and *Cucumis melo* L., uncovering widespread NLR variation and highlighting the limitations of reference genomes in capturing the full NLR repertoire of a species (Amas et al., 2023; Mo et al., 2024; Thatcher et al., 2023; Zhang et al., 2025; Zhuang et al., 2026). However, the variation and evolutionary dynamics of the pan‐NLRome across closely related species remain largely unexplored.

Rice (*Oryza sativa* L.), maize, and sorghum (*Sorghum bicolor* (L.) Moench) are closely related grass crops that serve as major sources of calories for human consumption. Their distinct pathogen spectra and domestication histories offer a natural model to investigate NLR conservation and variation. Leveraging the pan‐genomes recently developed for these three crops (Hufford et al., 2021; Qin et al., 2021; Tao et al., 2021), this study aims to (i) characterize and compare NLR repertoires in these three related crops, (ii) investigate the genetic variation and evolutionary dynamics of NLR genes, and (iii) assess the potential utility of NLRs by analyzing their co‐localization with disease resistance quantitative trait loci (QTLs). The findings from this study will enhance our understanding of NLR variation in these important crops and provide valuable targets for improving disease resistance.

## 2 METHODS

### 2.1 Identification and annotation of NLR genes

Genomic sequences from 75 assemblies were analyzed using NLR‐annotator (Steuernagel et al., 2020) to detect putative NLR loci. Each locus was subsequently extended by 50 kb on both flanking sides to define broader NLR‐associated genomic regions, ensuring the inclusion of complete gene models. Gene prediction and annotation within these regions were performed using BRAKER (Brůna et al., 2021), guided by multiple curated NLR protein reference datasets to enhance prediction accuracy and completeness (Calle García et al., 2022; Kourelis et al., 2021; D. Tang et al., 2022; Van De Weyer et al., 2019). Predicted gene models were then subjected to domain annotation using hmmscan (HMMER v3.3). Genes containing an NB‐ARC domain that overlapped with predefined NLR‐associated genomic regions were ultimately designated as NLRs.

### 2.2 Physical clustering of NLR genes

Clustering patterns of NLR genes in the three crops were assessed using the gene_cluster_identification module in GALEON. The maximum physical distance threshold used to define NLR clusters in each crop was calculated using the estimate_g_parameter module of Galeon (Pisarenco et al., 2024), resulting in *g* values of 30 kb for rice, 100 kb for sorghum, and 200 kb for maize. This data‐driven approach accounted for different genome sizes and gene densities in the three crops. A physical cluster was defined as a set of NLR genes in which pairwise distances between all members did not exceed the species‐specific g threshold and were not interrupted by unrelated clusters.

### 2.3 Pan‐NLRome analysis

NLR protein sequences from each crop were first extracted, to analyze species‐wise Pan‐NLRome. OGs were identified using OrthoFinder with default setting (Emms & Kelly, 2019). All NLR proteins from the 75 genomes were then pooled for a second OrthoFinder run to construct a cross‐species pan‐NLRome. OGs were categorized as core (present in ≥95% of genomes), shell (present in 5%–95%), or cloud (present in <5%), representing conserved, variable, and rare NLRs, respectively. Selection pressure was estimated using TBtools (Chen et al., 2023) via its Ka/Ks Calculator 2.0 module (calling KaKs_Calculator, model YN00), which computed pairwise Ka, Ks, and Ka/Ks values for each gene pair within an OG. Gene pairs with Ks = 0 or Ka or Ks > 2 were excluded, and the remaining values were summarized to yield representative Ka/Ks metrics for each OG, providing a quantitative basis for downstream analyses of NLR evolution and functional divergence.

### 2.4 Synteny analysis of NLR loci

Intra‐ and interspecific synteny analyses of NLR loci were performed using the Python implementation of the JCVI toolkit (H. Tang et al., 2024). Gene annotation files and protein sequences were standardized using the jcvi.formats.gff module, and representative transcripts were extracted. Pairwise BLASTP comparisons were executed to identify homologous gene pairs with an *E*‐value threshold of 1e‐5. Syntenic blocks were subsequently detected using the ortholog and dotplot submodules in jcvi.compara.catalog. The resulting syntenic relationships were visualized as genome‐wide dot plots to assess NLR collinearity, conservation, and structural rearrangements both within and between species.

### 2.5 Phylogenetic tree construction

Evolutionary relationships among the 75 cereal accessions were constructed as a genome‐wide species phylogeny based on a curated set of single‐copy orthologs. Protein sequences were aligned using MUSCLE v5.1 (Edgar, 2022) with default parameters. Aligned sequences were concatenated and used for maximum‐likelihood phylogenetic inference in IQ‐TREE v2.1.3 (Minh et al., 2020). Model selection was performed automatically (‐m MFP), and branch support was assessed with 1000 ultrafast bootstrap replicates (‐bb 1000).

### 2.6 Analysis of NLR copies within syntenic physical clusters

NLR copies within syntenic physical clusters was analyzed using homology‐based searches using reference NLR datasets from *Sorghum bicolor* (BT × 623), *Oryza sativa* (MSU), and *Zea mays* (B73). For each species, an NLR protein database was constructed and queried against itself using DIAMOND BLASTP (Buchfink et al., 2021). Self‐alignments were performed in sensitive mode with thresholds of sequence identity ≥50%, query and subject coverage ≥80%, and *E*‐value ≤1e‐5. No limit was imposed on the maximum number of target sequences to ensure exhaustive retrieval.

### 2.7 Analysis of TEs around NLR genes

Genomic sequences extending 2 kb upstream and downstream of each NLR gene were extracted using BEDTools v2.31 (Quinlan & Hall, 2010). These flanking sequences were annotated for TEs using EDTA (Extensive de novo TE Annotator) v2.0.1 (Ou et al., 2019), which identifies major TE superfamilies through structure‐ and homology‐based detection. Chi‐squared tests were applied to compare TE distributions near NLR loci with genome‐wide patterns.

### 2.8 Identification of resistance hotspots and co‐localization with NLRs

Disease‐resistance QTLs were obtained from the Sorghum QTL Atlas (https://aussorgm.org.au/sorghum‐qtl‐atlas) for sorghum and from Ensembl Plants (https://plants.ensembl.org) for rice, while resistance‐associated quantitative trait nucleotides (QTNs) in maize were compiled from 37 published GWASs (Tables S15 and S16). QTL hotspots were defined by merging overlapping intervals, with regions supported by at least two independent QTLs considered as hotspots (Yin et al., 2024). For QTNs, a sliding‐window approach was applied, and genomic windows of 0.8 Mb containing three or more significant SNPs were designated as hotspots. Genomic coordinates of hotspots were intersected with NLR positions to identify co‐localization events. To ensure consistency across genome assemblies, all coordinates were standardized using the LiftOver tool (Genovese et al., 2024).

## 3 RESULTS

### 3.1 Genome‐wide survey of NLRs across three grass crops

**FIGURE 1:**
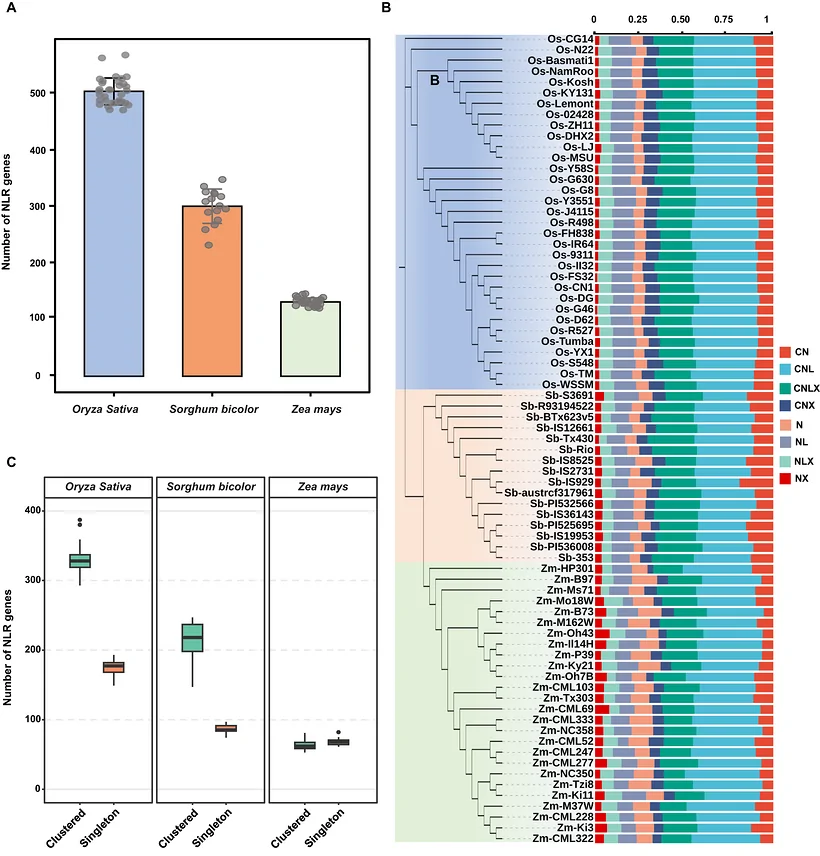
Nucleotide‐binding leucine‐rich repeat (NLR) abundance, composition, and domain profiles in three grasses. (A) NLR gene counts in three crops; bars show species means and dots individual genomes for *Oryza sativa*, *Sorghum bicolor*, and *Zea mays*. (B) Species tree and NLR subclass composition for each accession. (C) Numbers of clustered, singleton NLRs in three crops.

To investigate the genetic variation of NLRs among the three closely related grass crops, we performed a genome‐wide survey across 75 high‐quality assemblies, including 16 sorghum, 26 maize, and 33 rice, and identified 24,944 nonredundant NLR genes (Tables S1 and S2). The number of NLRs detected per genome was relatively consistent within each species, with rice exhibiting the greatest intra‐species variation, ranging from 473 to 570 genes (Figure 1A). However, marked interspecific differences were observed in total NLR gene number. Despite possessing the largest genome size, maize contained the fewest NLRs, averaging 132 genes per genome, whereas sorghum and rice harbored an average of 302 and 506 NLRs per genome, respectively (Figure 1A; Table S2).

All identified NLRs were classified into eight canonical domain‐architecture types (CN [coiled‐coil nucleotide‐binding], CNL, CNLX [coiled‐coil nucleotide‐binding leucine‐rich repeat with integrated domain], CNX [coiled‐coil nucleotide‐binding with integrated domain], N, NL, NLX [nucleotide‐binding leucine‐rich repeat with integrated domain], and NX [nucleotide‐binding with integrated domain]; “X” indicates the presence of an ID). Despite variation in total gene counts, the three cereals exhibited broadly comparable domain compositions, with the eight types representing similar proportions of the overall NLRome (Figure 1B). CNL and CNLX types, which encompass all three canonical domains, were the most abundant, jointly accounting for over 50% of the total NLRs in each crop. Domain types containing two of the three core domains (NL, NLX, CN, and CNX) collectively represented 31%–36% of the NLR complement. N and NX types, each containing only a single canonical domain, were the least frequent. NLRs carrying IDs (the four “X” types) were generally less abundant than their nonintegrated counterparts. Nevertheless, moderate interspecific variation was observed in the proportion of each type. For instance, CNLs comprised 35.41% of rice NLRs, but only 29.87% in sorghum. These results illustrate a largely conserved domain architecture across the three crops with moderate lineage‐specific diversification, potentially reflecting a shared evolutionary origin.

The chromosomal distribution of NLRs was highly uneven, with clear clustering patterns detected in all three genomes. We identified physical NLR clusters based on their genomic positions using a method that accounted for interspecies differences in genome size and gene density (Pisarenco et al., 2024). Among the three crops, maize exhibited the highest proportion of singleton NLRs (44.29% on average), whereas singletons comprised 28.76% and 34.71% of NLRs in sorghum and rice, respectively (Figure 1C). The clustering patterns of NLR genes may influence their evolutionary strategies, as NLR clusters serve as genomic hotspots for diversification and innovation (Kourelis & van der Hoorn, 2018). Notably, different NLR domain types displayed both conserved and species‐specific clustering patterns. CN and CNX types were significantly enriched in clusters across all three species, whereas CNLs were preferentially found as singletons (Chi‐squared test, *p* < 0.05; Table S3). CNLX genes were cluster‐enriched only in rice, while NLs showed significant clustering in rice and maize but not in sorghum.

### 3.2 Diversity of ID in NLR genes

**FIGURE 2:**
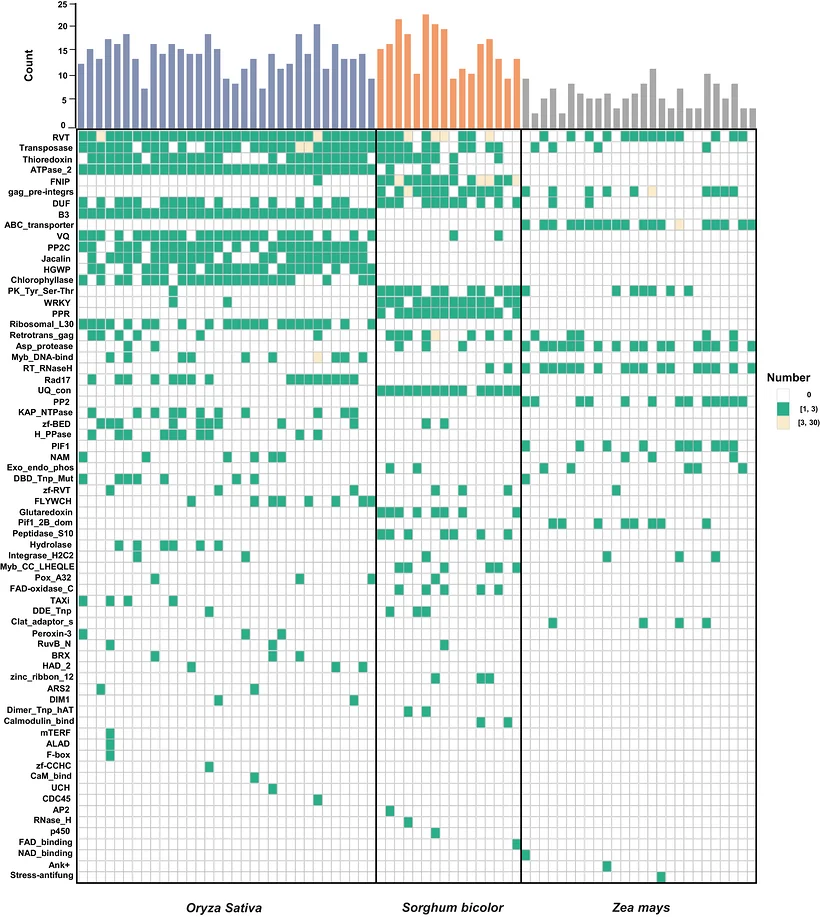
Integrated‐domain (ID) landscape of nucleotide‐binding leucine‐rich repeats (NLRs) in three crops. Each column represents an accession. Bar plot showing the number of NLRs with integrated domain.

IDs are key structural components of NLR proteins that mechanistically mediate direct effector recognition. Therefore, we further analyzed the diversity and distribution of IDs. In total, 906 of the 24,944 NLRs contained at least one ID, leading to the identification of 68 distinct ID types (Figure 2; Table S4). Rice exhibited a slightly lower proportion of ID‐containing NLRs (2.86%) compared with sorghum (5.29%) and maize (5.01%). Nevertheless, rice possessed the largest number of unique ID types (44), followed by sorghum (33) and maize (21). Eight ID types were shared among all three grass species (Figure 2; Table S5). Notably, five of these shared IDs, including reverse transcriptase (RVT), Retrotrans_gag, Integrase_H2C2, Transposase, and zf‐RVT, are derived from retrotransposons or DNA transposons, suggesting that transposable element (TE) activity may have facilitated the acquisition of exogenous domains by NLRs in grasses. In sorghum, ID‐NLRs were significantly enriched in singleton genes (Chi‐squared test, *p* < 0.05), whereas no such enrichment was observed in rice or maize.

Beyond these shared IDs, 25, 8, and 13 lineage‐specific IDs were identified in rice, maize, and sorghum, respectively (Figure 2; Table S4). These lineage‐restricted ID fusions likely reflect species‐specific genomic innovation driven by distinct pathogen pressures. Among these, a C‐terminal B3 DNA‐binding domain was consistently identified in NLRs from all 33 rice accessions. Fusion of the B3 domain to NLRs has been reported to enable coordinated pathogen recognition and transcriptional reprogramming of immune responses (Bailey et al., 2018; Kroj et al., 2016). In maize, the most prevalent lineage‐specific ID was the ABC_transporter domain, whose fusion with NLRs may couple pathogen perception with membrane transport or metabolite efflux to accelerate immune activation (Hwang et al., 2016). Collectively, the identification of these diverse IDs provides valuable clues for uncovering novel effector‐targeting mechanisms in grass NLRs.

### 3.3 The Pan‐NLRome in grass

To capture the variation of NLR genes among the three grass crops, we first clustered NLR proteins from multiple genomes into orthogroups (OGs) based on sequence similarity within each crop (Tables S2 and S5). A total of 573, 293, and 133 OGs were identified in rice, sorghum, and maize, respectively. Saturation analyses indicated that randomly selecting only six sorghum genomes (37.5%), 10 rice genomes (30.3%), or nine maize genomes (34.6%) could capture >95% of OGs within each species (Figure S1). This rapid saturation likely reflects the uneven distribution of genes among OGs, with >50% of all NLRs concentrated within approximately 30% of the largest OGs (Figure S2). Such skewed clustering underscores family‐oriented expansion of NLRs in grass genomes, suggesting that their overall repertoires are disproportionately shaped by large gene families. Notably, NLR genes experienced most expansion and were largely independent in the three crops with limited overlap among the large OGs.

**FIGURE 3:**
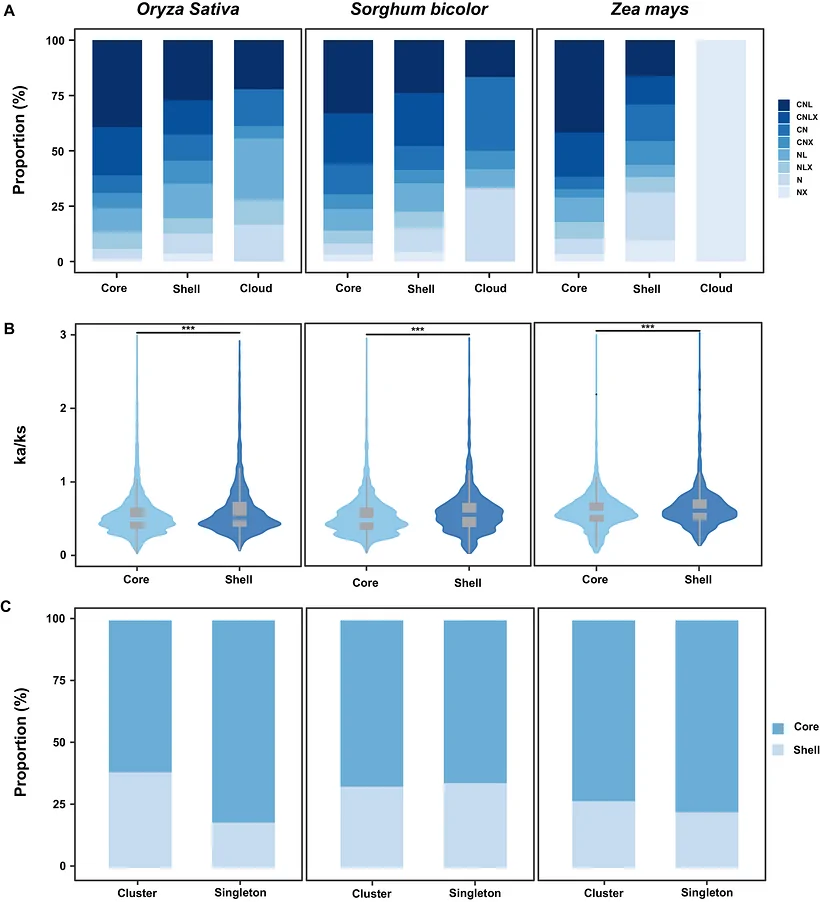
Comparison of Pan‐NLRome in three crops. (A) Composition of core, shell, and cloud genes. (B) Comparison of Ka/Ks values between core and shell nucleotide‐binding leucine‐rich repeats (NLRs). (C) Proportions of singleton and cluster NLR in core and shell genes. Cloud is omitted due to low counts.

OGs were further classified into core, shell, and cloud categories based on gene occupancy frequency across accessions (Figure S1). Comparable proportions of core, shell, and cloud OGs were observed among the three species, with core OGs contributing roughly 50% of the total OGs. Canonical CNL genes represented the major component of the core OGs in all three species, accounting for 39%, 33%, and 42% in rice, sorghum, and maize, respectively (Figure 3A). Sorghum exhibited the highest proportion of CNLX genes (23%) in core OGs, suggesting a greater reliance on fusion between CNLs and IDs to optimize effector recognition. Within the shell category, rice retained a predominance of canonical CNL genes, whereas maize showed a notable enrichment of simplified N‐type genes (Figure 3A). Sorghum maintained a balanced composition of CNL and CNLX genes within the shell subset. The composition of cloud OGs varied considerably among species, partly due to stochastic fluctuations arising from their small numbers. In rice and maize, singleton NLRs were significantly enriched in core OGs, whereas clustered NLRs were overrepresented in shell OGs (Figure 3C; Table S6). The distribution of ID‐NLRs in pan‐NLRome also differed across species. Both sorghum and maize showed significant enrichment of ID‐NLRs in the shell subset, whereas rice did not. Moreover, shell NLRs exhibited significantly higher Ka/Ks ratios than core genes, indicating that they evolve more rapidly (Figure 3B).

**FIGURE 4:**
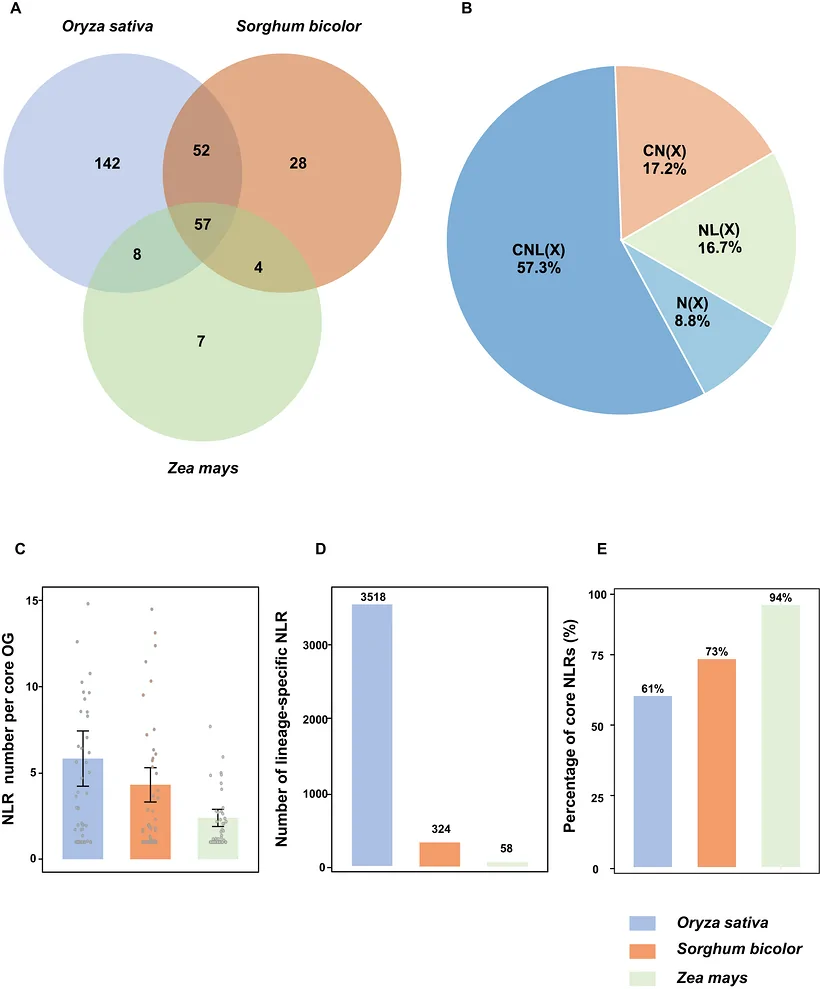
Cross‐species Pan‐NLRome of the three crops. (A) Cross‐species overlap of nucleotide‐binding leucine‐rich repeat (NLR) orthogroups. (B) Composition of the orthogroups (OGs) shared by all three species. (C) NLR number per shared OG. (D) Numbers of species‐specific NLR genes. (E) Proportion of shared OGs genes in NLR repertoires of the three crops.

We next performed cross‐species clustering of NLR proteins from rice, sorghum, and maize, identifying a total of 298 OGs. Among them, 57 OGs were conserved across all three crops, representing the core NLRome of grasses. These core OGs contained 16,517 genes, accounting for approximately 66% of all identified NLRs (Figure 4A; Table S7). Approximately 60% of these core OGs belonged to the CNL/CNLX subclass, reflecting the extensive expansion and evolutionary conservation of this NLR type in grasses (Figure 4B). Comparison of cross‐species pan‐NLRome showed distinct NLR repertoire in the three crops (Figure 4E). Maize exhibited the most conserved NLR repertoire, with 94% of its NLRs assigned to core OGs and only 58 genes (2%) classified as maize‐specific. In contrast, rice displayed the most extensive lineage‐specific expansion, with rice‐specific OGs comprising 21% of its NLRs, while core OGs accounted for 61%. Sorghum showed an intermediate pattern, with approximately 75% of its NLRs belonging to core OGs and 7% being sorghum‐specific. Consistent with their dynamic nature, ID‐NLRs were preferentially enriched in the shell OGs.

Within the core OGs, rice exhibited a higher average NLR number per OG (5.86) than sorghum (4.33) and maize (2.41) (Figure 4C). Rice also harbored the largest number of lineage‐specific NLRs among the three species (Figure 4D). Taken together, these findings suggest that the elevated NLR count in rice results from both amplification of core OGs and expansion of lineage‐specific genes. Conversely, maize displayed minimal expansion of both core and lineage‐specific NLRs, resulting in a structurally compact NLR repertoire. These contrasting patterns likely reflect divergent evolutionary trajectories and distinct pathogen pressures among the three crops.

### 3.4 Structural variation and evolutionary dynamics of NLR genes

**FIGURE 5:**
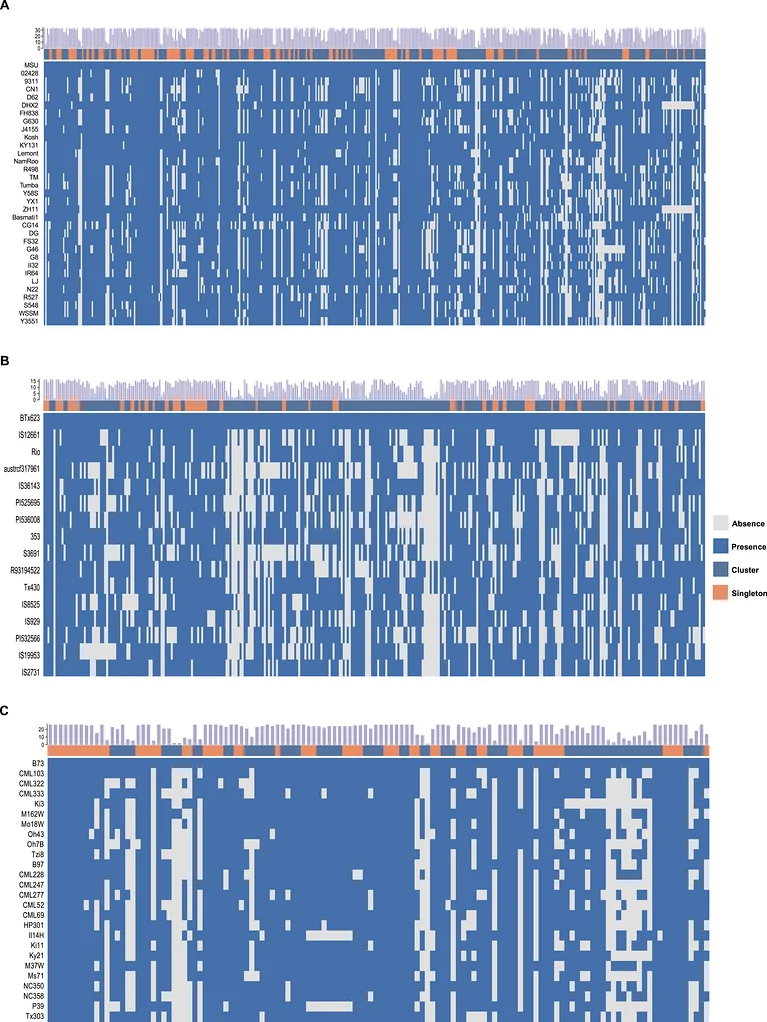
Presence/absence patterns of syntenic nucleotide‐binding leucine‐rich repeats (NLRs) in rice (A), sorghum (B), and maize (C). In each panel, columns represent syntenic NLR loci defined relative to the corresponding reference genome, and rows represent individual genomes within the species. Blue indicates presence and gray indicates absence. The bar plot at the top shows the number of genomes in which each syntenic NLR is detected. The annotation track below indicates NLR type, with orange representing singleton NLRs and light blue representing clustered NLRs.

To investigate the structural variation and evolutionary trajectories of NLR genes, we conducted intra‐ and interspecific comparisons to construct a synteny‐based presence–absence variation (PAV) matrix (Tables S8 and S9). Overall, >56% of NLR genes exhibited PAV within each crop, with sorghum showing the highest proportion (∼58.1%) (Figure 5; Figures S3–S5). Notably, over 58% of clustered NLRs displayed PAV in all three species, indicating a significant enrichment of PAV in clustered NLRs (Chi‐squared test, *p* < 0.01). In contrast, singleton NLRs were significantly underrepresented in the PAV category, with only about half showing PAV (Chi‐squared test, *p* < 0.01). The higher PAV frequency of NLRs in sorghum compared with rice and maize is likely related to its greater proportion of clustered NLRs.

Interspecific analyses revealed substantial conservation of NLR synteny among rice, sorghum, and maize, with approximately 40% of NLRs retaining a syntenic counterpart in at least one other species (Table S10). A smaller proportion of syntenic NLRs was detected in rice (32%) compared with sorghum (47%) and maize (57%), reflecting the closer phylogenetic relationship between sorghum and maize. Marked differences in NLR number were observed across syntenic regions of the three genomes. For instance, the corresponding syntenic intervals on rice Chr11 (6.5–6.8 Mb), sorghum Chr05 (14.4–15.3 Mb), and maize Chr04 (209.4–209.8 Mb) harbored 13, 8, and 3 NLRs, respectively.

TEs are known to be major drivers of genomic PAV (Tao et al., 2019). To evaluate the potential role of TEs in shaping NLR PAV, we analyzed TE composition within the 2‐kb flanking regions of NLR genes. Across all three crops, PAV‐associated NLRs contained a higher proportion of TE sequences than their non‐PAV counterparts (Table S11). We further classified TEs into 15 subclasses according to their transposition mechanisms and sequence characteristics. Notably, long terminal repeat (LTR)/Copia elements were significantly enriched near PAV NLR genes in sorghum (Fisher's exact test, *p* < 0.05), a pattern consistent for both clustered and singleton NLRs (Table S12). In rice, LTR/Gypsy elements were preferentially enriched in the flanking regions of PAV NLRs (Chi‐squared test, *p* < 0.05), with enrichment driven primarily by singleton NLRs. Collectively, these results support that TEs contribute to the generation and maintenance of PAV NLR in grasses, with distinct TE classes exerting species‐specific influences on NLR genomic dynamics.

### 3.5 Co‐localization analysis with disease‐resistance loci

**FIGURE 6:**
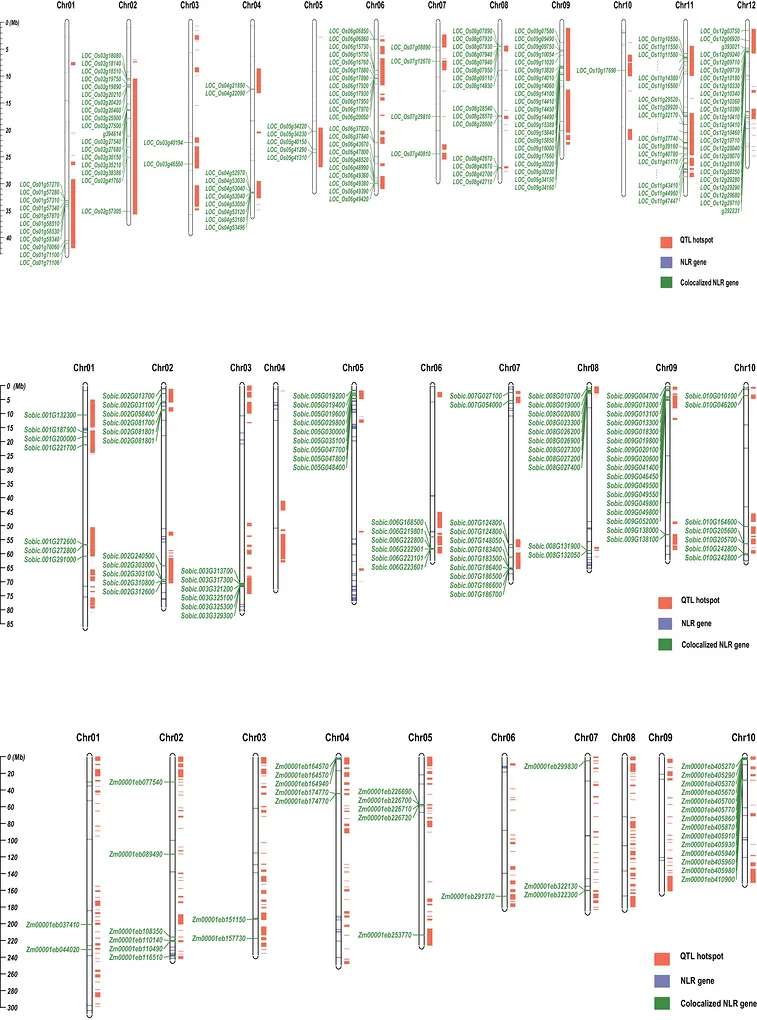
Co‐localization of nucleotide‐binding leucine‐rich repeat (NLR) genes with disease resistance quantitative trait locus (QTL) hotspots.

To evaluate the potential impact of NLR genes on disease‐resistance traits, we performed a co‐localization analysis between NLR genes and previously reported disease‐resistance QTLs in rice, maize, and sorghum. QTLs and genome‐wide association study (GWAS) signals associated with disease‐resistance traits were compiled from published studies for the three species, and resistance hotspots were delineated following the approach described by Yin et al. (2024). In total, 413 QTLs, 5801 significant single‐nucleotide polymorphisms (SNPs), and 604 QTLs were collected for rice, maize, and sorghum, respectively, resulting in 81, 726, and 104 resistance hotspots (Table S13). Among them, a total of 21 synthetic disease resistance QTL hotspots were found across crops, including *Rp1* loci. Co‐localization analysis revealed that 47.6% of rice NLRs, 34.4% of maize NLRs, and 26.9% of sorghum NLRs were located within these resistance hotspots (Figure 6), indicating significant enrichment across all three crops. Furthermore, clustered NLRs were more frequently located within resistance hotspot regions than singleton NLRs, which could be due to their diverse nature.

**FIGURE 7:**
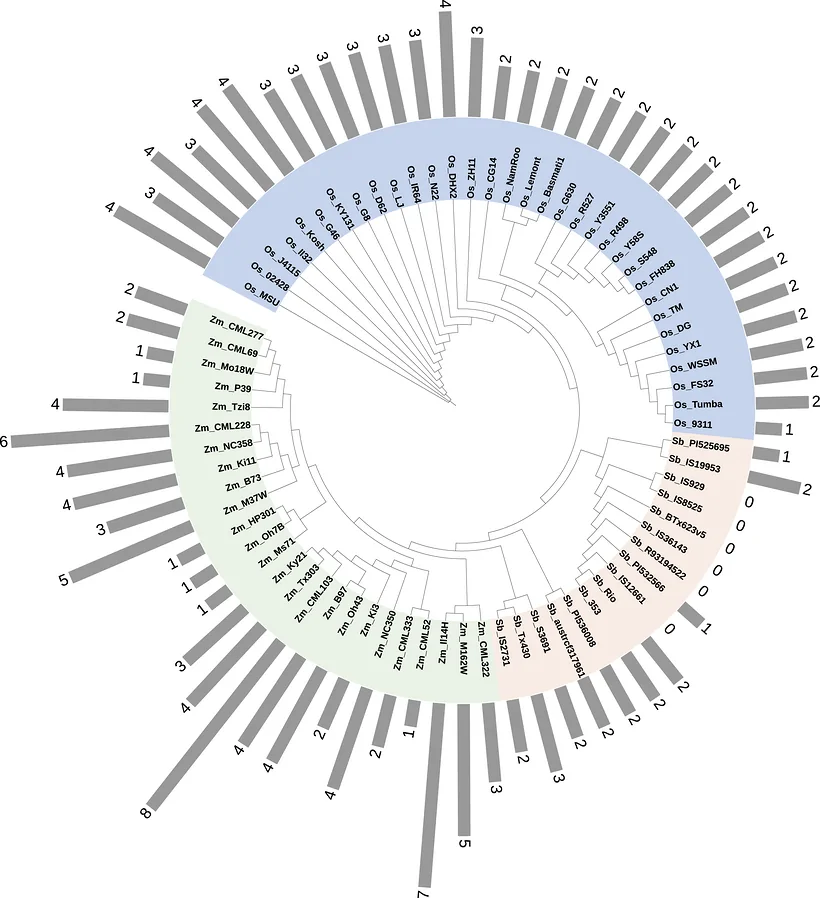
Phylogeny and copy‐number variation of *Rp1‐D* homologs.

In maize, a prominent resistance QTL hotspot on chromosome 10 encompassed multiple loci conferring resistance to several diseases, including common leaf rust, northern leaf blight, and southern leaf blight. Within this hotspot, a cluster of *Rp1‐D* genes, known to confer race‐specific resistance to *Puccinia sorghi* and associated with resistance to Goss's wilt, were identified (Collins et al., 1999). The copy number of *Rp1‐D* homologues varied from one to eight among the 26 maize genomes. As these *Rp1‐D* copies share >90% sequence similarity, the observed variation may reflect potential dosage effects, although functional diversification among paralogous copies cannot be excluded. Interestingly, this *Rp1‐D* cluster in maize was syntenic to an NLR cluster (*Sobic.008G027300*, *Sobic.008G027200*, and *Sobic.008G027400*) with an averaged 90% sequence similarity on sorghum chromosome 8, which co‐localized with a resistance hotspot associated with rust and other foliar diseases (Table S14). This observation suggests a conserved rust‐resistance mechanism between maize and sorghum. The sorghum *SbRp1‐D* displayed copy number variation in the sorghum pangenome (Figure S5). In rice, homologs of *Rp1‐D* (*LOC_Os01g57270*, *LOC_Os01g57280*, *LOC_Os01g57310*, and *LOC_Os01g57340*) with an averaged 90% sequence similarity were identified on chromosome 1 with copy number variation across genomes (Figure 7). However, three of the *Rp1‐D* homologs, *LOC_Os01g57280* (*Pi64*), *LOC_Os01g57310* (*Pi37*), and *LOC_Os01g57340* (*Pish*), confer blast resistance in rice (Lin et al., 2007; Ma et al., 2015; Takahashi et al., 2010). Together, these co‐localization results underscore the critical role of NLR genes in mediating disease resistance and highlight key candidate genes for future functional characterization and crop improvement.

## 4 DISCUSSION

In this study, we conducted a comprehensive analysis of NLR repertoires across 75 genomes from three major cereal crops and uncovered pronounced intra‐ and interspecific variation in NLR composition and genomic organization. Despite substantial differences in NLR gene number, the three crops exhibited largely conserved domain architectures and pan‐NLRome profiles, with strong lineage‐restricted expansion of NLR gene families. Nevertheless, divergent evolutionary trajectories were evident among them, with distinct proportions of common gene and lineage‐specific genes in the three crops. Extensive PAV of NLR genes was detected in all three crops, with an enrichment of clustered NLRs. Notably, NLR genes, particularly cluster ones, were enriched in resistance QTL hotspots, suggesting their critical contribution to disease resistance. Collectively, these results highlight both conserved patterns and lineage‐specific diversification of NLRs across the three globally important cereal crops, providing a valuable genomic resource for crop improvement.

The number of NLR genes varies markedly among plant species (E. Baggs et al., 2017). Recent large‐scale analyses have revealed convergent NLR reduction in angiosperms with specialized ecological niches or reduced pathogen exposure (Liu et al., 2021; Ngou et al., 2022). Such convergence underscores the influence of ecological context on NLR repertoire size. Consistently, the divergent NLR gene counts among rice, maize, and sorghum observed in this study likely reflect lineage‐specific immune strategies shaped by distinct habitats and pathogen pressures. The relatively smaller canonical NLR repertoire in maize may be one of the factors contributing to its greater reliance on quantitative resistance. Despite quantitative differences, NLRs maintained comparable domain architectures across the three species, indicating potential evolutionary constraints on NLR structural organization. The elevated NLR copy number in rice appears to result from both the expansion of common genes and amplification of lineage‐specific genes. Clear clustering patterns of NLR genes were detected in all three species, consistent with previous reports (Michelmore & Meyers, 1998). However, NLR clustering patterns varied across species and domain types, with sorghum and the CN/CNX domain types exhibiting the highest proportion of NLR clusters. The varying content of NLR clusters likely reflects different evolutionary forces driving their formation, including tandem duplication, unequal crossing‐over, ectopic recombination, and gene conversion (Barragan & Weigel, 2021; Michelmore & Meyers, 1998).

Integration of exogenous domains (IDs) provides an efficient mechanism for functional innovation within NLRs. It introduces new recognition or signaling modules, enabling plants to rapidly diversify immune capacity during long‐term coevolution with pathogens. Previous studies in *Arabidopsis* reported that approximately 10%–15% of NLRs harbor IDs, with around 50 distinct ID types identified (Barragan & Weigel, 2021; Van De Weyer et al., 2019). In contrast, our analysis detected fewer than 6% of NLRs with IDs across the three grass crops, suggesting a lower frequency of domain integration compared to *Arabidopsis*. Nevertheless, we identified 68 distinct ID types, including several not previously reported in dicots. Eight ID types shared among the three crops were primarily associated with TE, indicating that TE‐mediated domain fusion represents a conserved mechanism of NLR diversification in grasses. Conversely, lineage‐specific IDs, such as B3‐NLRs in rice, ABC‐NLRs in maize, and PPR‐NLRs in sorghum, reflect independent functional innovations. Moreover, the enrichment of ID‐NLRs within the shell fraction of the pan‐NLRome highlights their likely role in environmental adaptation and evolutionary plasticity.

Divergent evolutionary trajectories of NLRs among the three cereals were revealed in this study. Rice exhibits both the highest copy number of conserved genes and the largest number of lineage‐specific NLRs, along with the most diverse set of ID types, reflecting genomic innovation to cope with persistent high disease pressure under humid environments. Many of these duplicated genes are physically clustered and display PAV, consistent with previous reports of structural expansion in rice (Qin et al., 2021). Sorghum maintains an intermediate level of common and lineage‐specific genes but exhibits the highest fraction of clustered loci and structural variation, indicating an evolutionary strategy that may rely more on the rearrangement and diversification of existing NLR loci. This pattern is exemplified by recent studies of sorghum anthracnose resistance genes, where both *ARG4* and *ARG5* were identified within clusters of duplicate NLR genes (Habte et al., 2024). In contrast, maize displays the most compact and conserved NLR repertoire, with limited lineage‐specific expansion. However, highly variable NLRs in maize have been reported, concentrating in four major subfamilies (Prigozhin et al., 2025). In contrast, a more widespread pattern of high NLR diversity has been observed in rice (Wang et al., 2025). However, NLRs represent only one component of the plant immune system. Thus, integrating broader immune signaling and disease history will be essential to fully interpret NLR evolution.

Our study found a substantial proportion of NLR genes overlapped with reported disease‐resistance QTL hotspots, supporting their critical role in crop protection. This is consistent with a previous report in *Brassica rapa* (Amas et al., 2023). Clusters of *Rp1‐D* and its homologs in maize and sorghum co‐localized with rust resistance QTLs, suggesting a conserved role of *Rp1‐D* in rust resistance. However, the co‐localization of *Rp1‐D* homologs with blast‐resistance QTLs in rice, along with the association of the maize *rp1* region with resistance to multiple diseases, may indicate functional diversification within this NLR gene cluster. The role of *Rp1‐D* homologs in rust and blast resistance in rice warrants further investigation. Altogether, the comparative framework established in this study provides a powerful genomic foundation for mining and utilizing NLR diversity to enhance disease resistance in cereal crops.

It is worth noting that our analysis of NLR genes may be influenced by genome assembly quality, particularly in highly repetitive regions such as the maize *rp1* locus, where accurate assembly remains challenging. The genomes used in this study were derived from three previous publications with varying assembly quality. Therefore, the results should be interpreted with caution, as assembly quality may affect NLR copy number estimates, cluster boundary definitions, and clustering status. In addition, our annotation of NLR genes using NLR‐annotator may be biased against noncanonical NLRs, for example, ADR1 (Steuernagel et al., 2020), and sequence‐based thresholds may not fully capture structural and functional similarity. Nevertheless, these limitations are more likely to impact fine‐scale structural resolution in specific regions rather than alter the overall patterns observed in this study.

In conclusion, this study analyzed the genetic diversity of 24,944 NLR genes across 75 genomes representing three grass crops and revealed both conserved structural features and divergent evolutionary trajectories among them. By integrating NLR gene profiles with structural variation and resistance QTL data, our results provide comprehensive insights into the evolution and functional diversification of NLRs in grasses and offer a valuable genomic resource for breeding disease‐resistant crop varieties.

## AUTHOR CONTRIBUTIONS

**Yanbo Wang**: Conceptualization; data curation; methodology; software; writing—original draft. **Tashi Dorjee**: Conceptualization; data curation; methodology; software; validation; visualization; writing—original draft. **Yinzi Wang**: Data curation; validation. **Di Peng**: Data curation; methodology; resources. **Lang Chen**: Data curation; resources; validation. **Chuanzheng Wei**: Data curation; methodology; resources. **Shichao Sun**: Data curation; methodology; visualization. **Mingyu Duan**: Data curation. **Hui Li**: Data curation. **Adrian Hathorn**: Data curation; software. **Emma Mace**: Writing—review and editing. **David Jordan**: Writing—review and editing. **Xun Wu**: Data curation. **Xiaocui Chen**: Writing—review and editing. **Yongfu Tao**: Conceptualization; funding acquisition; supervision; writing—review and editing.

## CONFLICT OF INTEREST STATEMENT

The authors declare no conflicts of interest.

## Supporting information




**Table S1** Summary of genome assemblies and NLR annotation statistics for the rice, maize, and sorghum accessions.
**Table S2** NLR repertoires of three grass crops.
**Table S3** Enrichment analysis of NLR domain types in cluster and singleton genes.
**Table S4** Distribution of integrated domains in three crops.
**Table S5** Overview of orthogroup assignment in three crops.
**Table S6** Enrichement analysis of pan‐NLRome category in clustered and signleton NLR genes.
**Table S7** Overview of cross‐species orthogroup assignment.
**Table S8** Mapping of pan‐NLR gene IDs to reference genome gene IDs in rice, sorghum, and maize.
**Table S9** PAV of NLR genes in three crops.
**Table S10** Syntenic relationships of NLR genes across the three crops.
**Table S11** TE annotation near NLR genes in the reference genomes of three crops.
**Table S12** Enrichment analysis of transposable elements around PAV NLR genes.
**Table S13** Disease resistance QTL hotspot regions in three crops.
**Table S14** Similarity of Rp1‐D homologous sequences identified in 26 maize genomes.
**Table S15** List of disease‐resistance‐associated QTNs in maize.
**Table S16** List of references for collecting maize disease resistance QTN.

**Figure S1** Pan‐NLRome of the three crops. A, *Sorghum bicolor*, orthogroup accumulation curves (left), proportions of core/shell/cloud orthogroups (inset), and presence‐absence matrix across accessions (right). B, *Zea mays*. C, *Oryza sativa*.

**Figure S2** Lorenz curves of NLR OG sizes in three grasses.

**Figure S3** Genome‐wide synteny of NLR loci across 33 rice genomes.

**Figure S4** Genome‐wide synteny of NLR loci across 16 sorghum genomes.

**Figure S5** Genome‐wide synteny of NLR loci across 26 maize genomes.

## Data Availability

The rice genome assemblies used in this study were obtained from the Rice Resource Center (https://ricerc.sicau.edu.cn), the maize genome assemblies were obtained from MaizeGDB (https://www.maizegdb.org), and the sorghum genome assemblies were obtained from the Phytozome database (https://phytozome‐next.jgi.doe.gov). All data generated and/or analyzed during this study are included in this published article and its supplementary information files.
