# Supplementary material for: Pan‐NLRome analysis uncovers genetic diversity and evolutionary dynamics among rice, maize and sorghum
Source: Plant Genome. 2026 May 23;19(2):e70257. doi: 10.1002/tpg2.70257 (PMC13492628; doi:10.1002/tpg2.70257)
Supplement: Supplementary file 1 [file TPG2-19-e70257-s001.pdf]

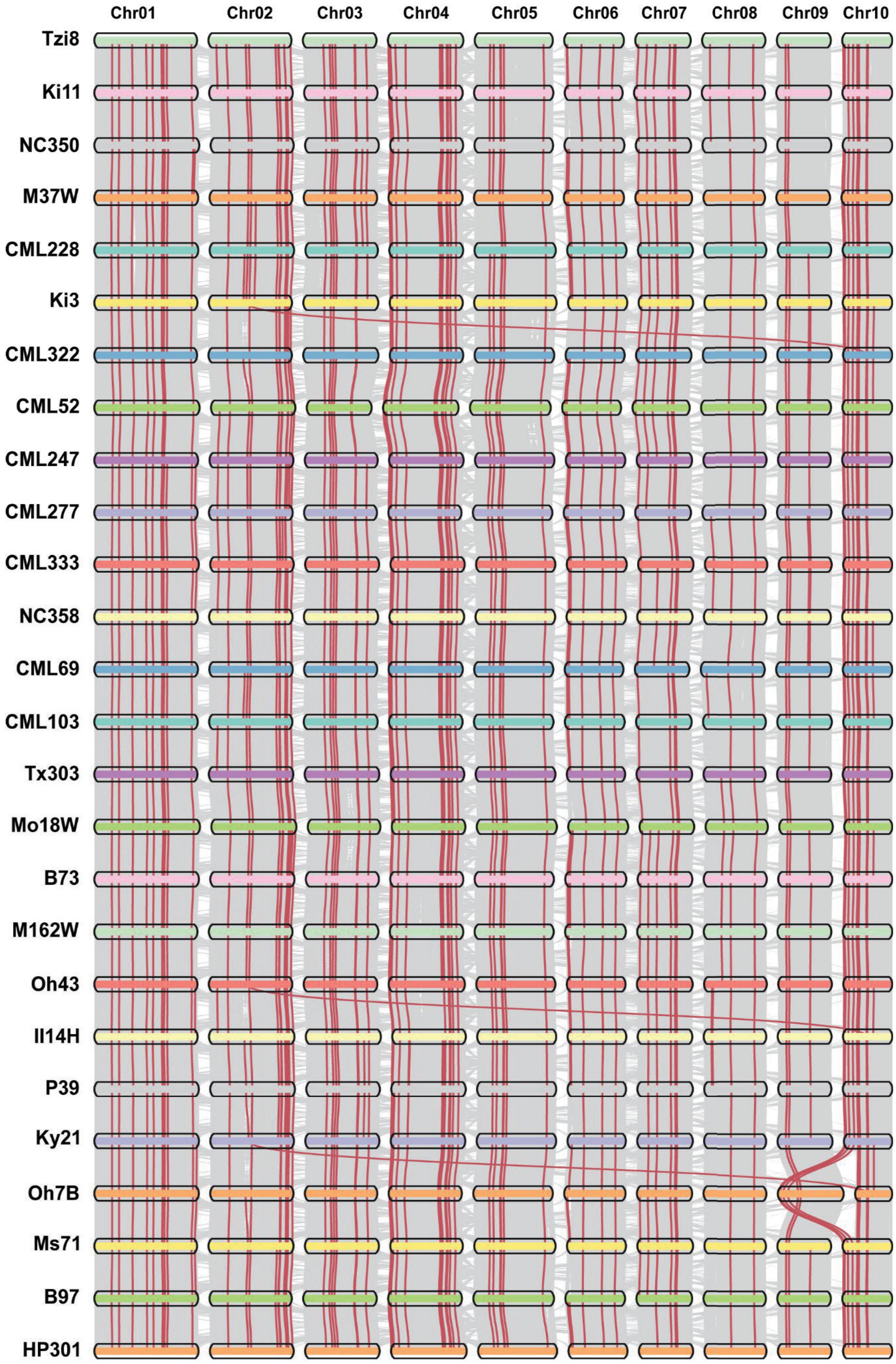
Chr01
Chr02
Chr03
Chr04
Chr05
Chr06
Chr07
Chr08
Chr09
Chr10
Tzi8
Ki11
NC350
M37W
CML228
Ki3
CML322
CML52
CML247
CML277
CML333
NC358
CML69
CML103
Tx303
Mo18W
B73
M162W
Oh43
Il14H
P39
Ky21
Oh7B
Ms71
B97
HP301
