# Supplementary material for: Pan‐NLRome analysis uncovers genetic diversity and evolutionary dynamics among rice, maize and sorghum
Source: Plant Genome. 2026 May 23;19(2):e70257. doi: 10.1002/tpg2.70257 (PMC13492628; doi:10.1002/tpg2.70257)
Supplement: Supplementary file 2 [file TPG2-19-e70257-s002.pdf]

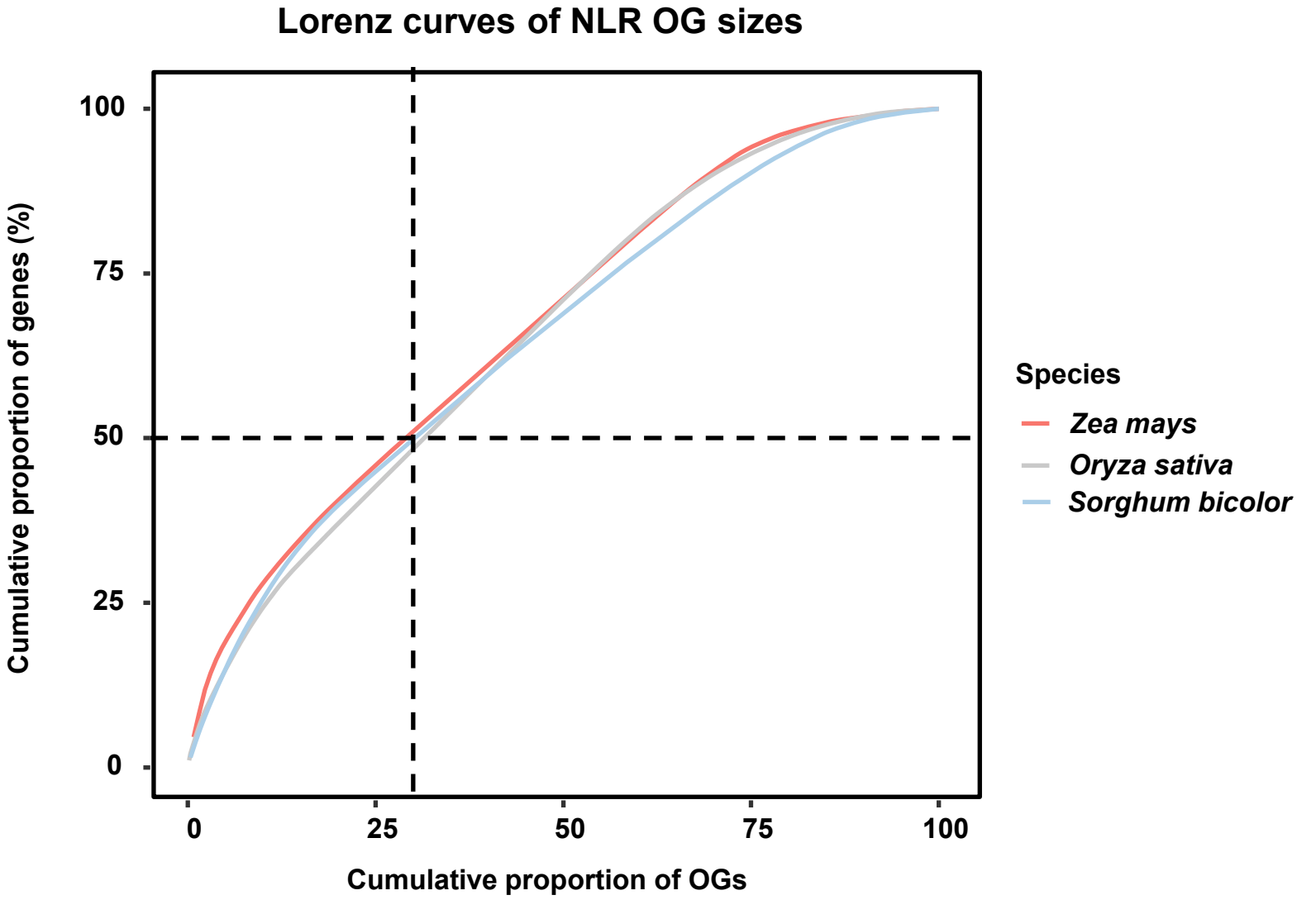
Lorenz curves of NLR OG sizes
Cumulative proportion of genes (%)
100
75
50
25
0
0
25
50
75
100
Cumulative proportion of OGs
Species
Zea mays
Oryza sativa
Sorghum bicolor
