# Supplementary material for: Pan‐NLRome analysis uncovers genetic diversity and evolutionary dynamics among rice, maize and sorghum
Source: Plant Genome. 2026 May 23;19(2):e70257. doi: 10.1002/tpg2.70257 (PMC13492628; doi:10.1002/tpg2.70257)
Supplement: Supplementary file 3 [file TPG2-19-e70257-s003.pdf]

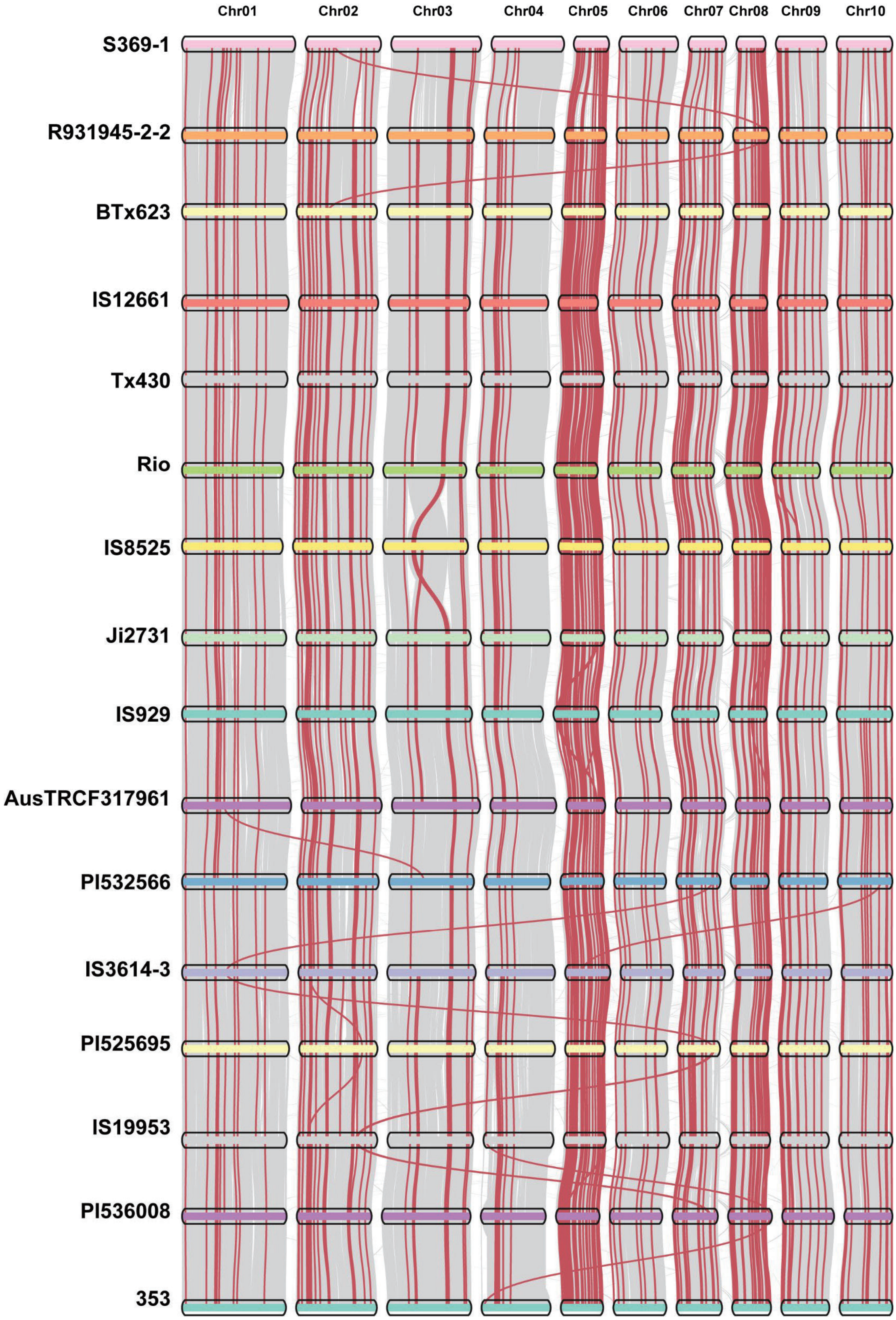

Chr01
Chr02
Chr03
Chr04
Chr05
Chr06
Chr07
Chr08
Chr09
Chr10
S369-1
R931945-2-2
BTx623
IS12661
Tx430
Rio
IS8525
Ji2731
IS929
AusTRCF317961
PI532566
IS3614-3
PI525695
IS19953
PI536008
353
