# Supplementary material for: Pan‐NLRome analysis uncovers genetic diversity and evolutionary dynamics among rice, maize and sorghum
Source: Plant Genome. 2026 May 23;19(2):e70257. doi: 10.1002/tpg2.70257 (PMC13492628; doi:10.1002/tpg2.70257)
Supplement: Supplementary file 4 [file TPG2-19-e70257-s004.pdf]

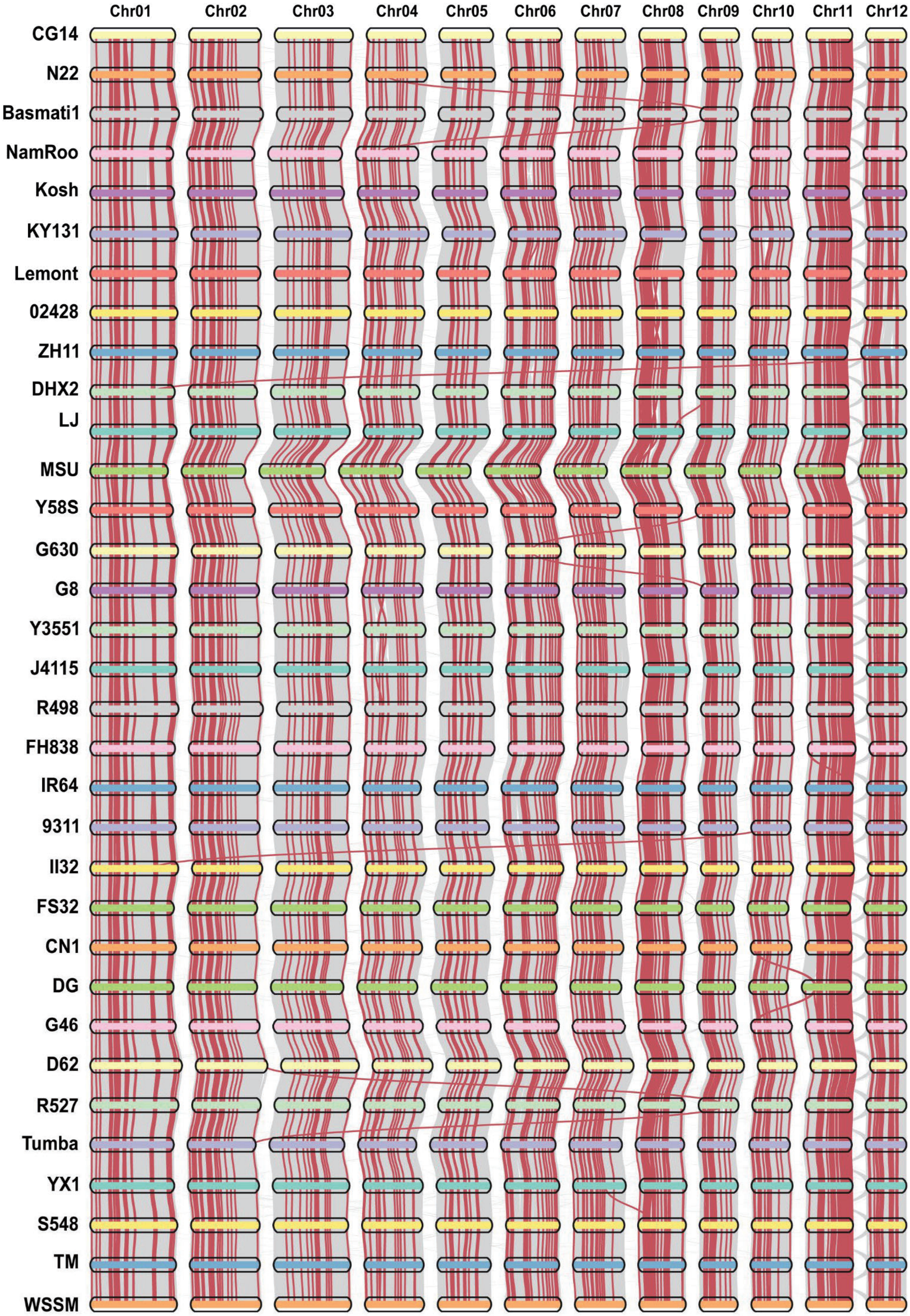
Chr01
Chr02
Chr03
Chr04
Chr05
Chr06
Chr07
Chr08
Chr09
Chr10
Chr11
Chr12
CG14
N22
Basmati1
NamRoo
Kosh
KY131
Lemont
02428
ZH11
DHX2
LJ
MSU
Y58S
G630
G8
Y3551
J4115
R498
FH838
IR64
9311
II32
FS32
CN1
DG
G46
D62
R527
Tumba
YX1
S548
TM
WSSM
