# Supplementary material for: Pan‐NLRome analysis uncovers genetic diversity and evolutionary dynamics among rice, maize and sorghum
Source: Plant Genome. 2026 May 23;19(2):e70257. doi: 10.1002/tpg2.70257 (PMC13492628; doi:10.1002/tpg2.70257)
Supplement: Supplementary file 5 [file TPG2-19-e70257-s006.pdf]

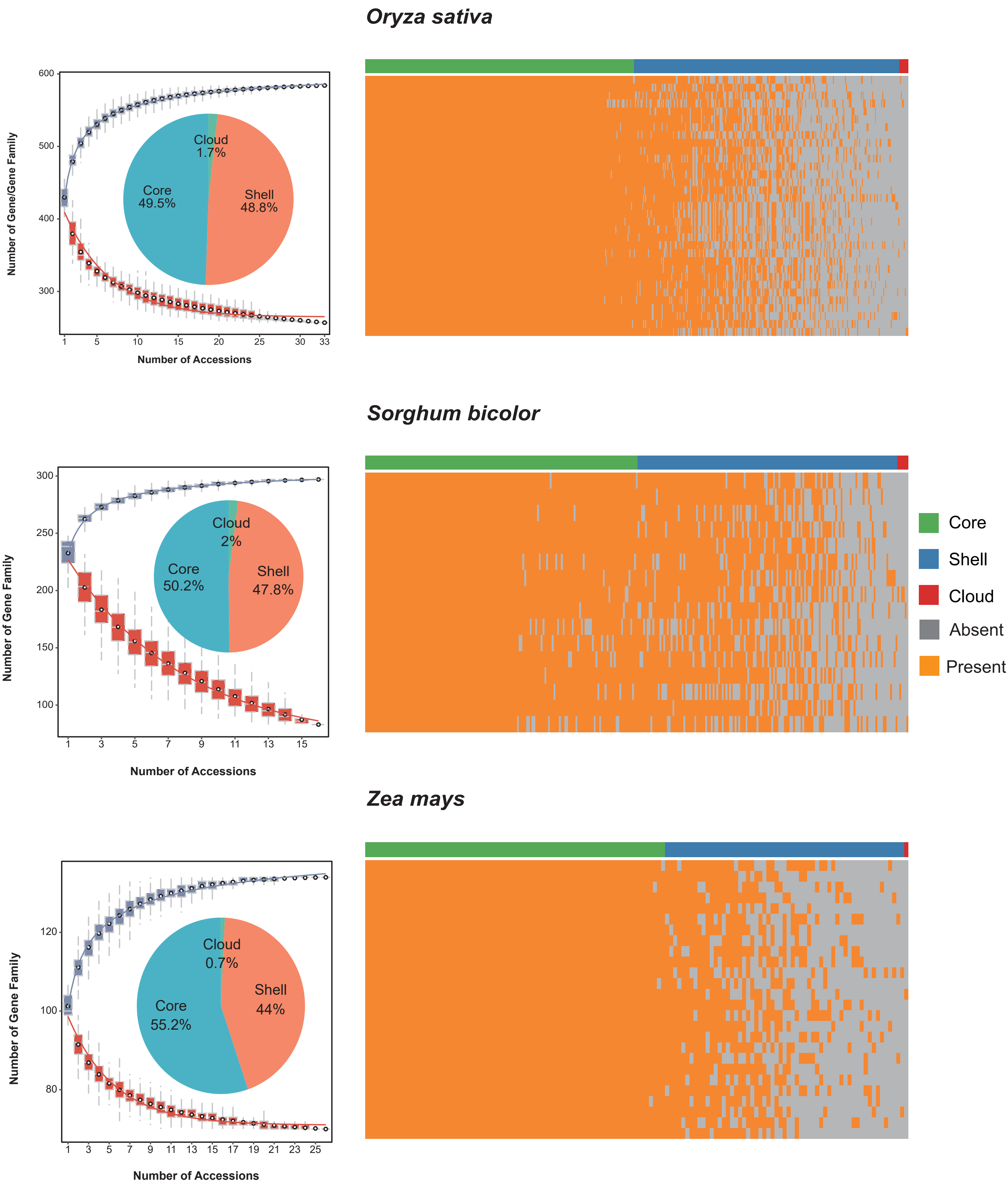
Oryza sativa
Number of Gene/Gene Family
600
500
400
300
1
5
10
15
20
25
30
33
Number of Accessions
Cloud
1.7%
Core
49.5%
Shell
48.8%
Sorghum bicolor
Number of Gene Family
300
250
200
150
100
1
3
5
7
9
11
13
15
Number of Accessions
Cloud
2%
Core
50.2%
Shell
47.8%
Core
Shell
Cloud
Absent
Present
Zea mays
Number of Gene Family
120
100
80
1
3
5
7
9
11
13
15
17
19
21
23
25
Number of Accessions
Cloud
0.7%
Core
55.2%
Shell
44%
